# Brain donation in psychiatry: results of a Dutch prospective donor program among psychiatric cohort participants

**DOI:** 10.1186/s12888-017-1513-x

**Published:** 2017-10-20

**Authors:** Geertje M. de Lange, Marleen Rademaker, Marco P. Boks, Saskia J. M. C. Palmen

**Affiliations:** 1 Netherlands Brain Bank, Netherlands Institute for Neuroscience, Meibergdreef 47, 1105 BA Amsterdam, the Netherlands; 2 Brain Center Rudolf Magnus, University Medical Center Utrecht, Utrecht University, PO Box 85500, 3508 GA Utrecht, the Netherlands

**Keywords:** Brain bank, Psychiatry, Post-mortem, Registration rates, Recruitment, Prospective brain-donor program

## Abstract

**Background:**

Human brain tissue is crucial to study the molecular and cellular basis of psychiatric disorders. However, the current availability of human brain tissue is inadequate. Therefore, the Netherlands Brain Bank initiated a program in which almost 4.000 participants of 15 large Dutch psychiatric research cohorts were asked to register as prospective brain donors.

**Methods:**

We approached patients with schizophrenia, bipolar disorder, major depressive disorder, obsessive-compulsive disorder, post-traumatic stress disorder, families with a child with autism or Attention Deficit Hyperactivity Disorder, healthy relatives and healthy unrelated controls, either face-to-face or by post. We investigated whether diagnosis, method of approach, age, and gender were related to the likelihood of brain-donor registration.

**Results:**

We found a striking difference in registration efficiency between the diagnosis groups. Patients with bipolar disorder and healthy relatives registered most often (25% respectively 17%), followed by unrelated controls (8%) and patients with major depressive disorder, post-traumatic stress disorder, and obsessive-compulsive disorder (9%, 6% resp. 5%). A face-to-face approach was 1.3 times more effective than a postal approach and the likelihood of registering as brain donor significantly increased with age. Gender did not make a difference.

**Conclusions:**

Between 2013 and 2016, our prospective brain-donor program for psychiatry resulted in an almost eightfold increase (from 149 to 1149) in the number of registered psychiatric patients at the Netherlands Brain Bank. Based on our results we recommend, when starting a prospective brain donor program in psychiatric patients, to focus on face to face recruitment of people in their sixties or older.

## Background

The use of human brain tissue is the main strategy to study the molecular and cellular basis of psychiatric disorders. However, the current availability is far from sufficient: 69% of the post-mortem studies on bipolar disorder in the last 30 years used material from a single source consisting of merely 50 cases (Stanley Foundation Collection) and for the second most widely used collection (Harvard), 80% of studies were conducted with the same 72 cases [1]. Increasing the number of available post-mortem brains of extensively phenotyped patients will facilitate post-mortem research, and is key in improving understanding of the pathophysiology of psychiatric disorders. Ultimately, this should lead to improved treatment strategies, alleviating the personal, social, and economic burden of psychiatric disorders [2, 3]. Therefore, the Netherlands Brain Bank (NBB) initiated the first worldwide prospective donor program for seven psychiatric disorders (NBB-Psy). Its aim is to establish a resource of well documented and high-quality brain tissue of patients diagnosed with schizophrenia (SCZ), bipolar disorder (BD), major depressive disorder (MDD), obsessive-compulsive disorder (OCD), post-traumatic stress disorder (PTSD), autism spectrum disorder (ASD) and Attention Deficit Hyperactivity Disorder (ADHD), non-psychiatric relatives and unrelated control subjects. To achieve this, NBB-Psy, in close collaboration with five Dutch university medical centers, actively approached extensively phenotyped participants of fifteen large psychiatric research cohorts to register as prospective brain donor at the NBB. We here present an interim report of the registration percentages of patients, relatives and unrelated control subjects, taking into account the effects of diagnosis, method of approach, age, and gender.

We hypothesized registration rates of 15-20%, which was based on our experience with approaching cohort patients with neurological disorders where the registration rates were 20 to 25%. We expected the registration rates in psychiatry to be somewhat lower than in neurology. In neurological disorders confirming the diagnosis post-mortem is an additional motivational factor [4]. Moreover, patients are on average older, and their disease has a clear neurodegenerative nature, which confronts patients with increasing constraints on their daily function and may thus motivate to contribute to scientific studies to eradicate the disorder they suffer from. We hypothesized that older people would be more willing to register [5], and that a face-to-face approach would be more effective than a postal approach. Finally, we hypothesized that gender [5] and diagnosis would not affect registration rate.

## Methods

### Study design and participants

Our study was a quantitative descriptive study of participants of fifteen psychiatric research cohorts in the Netherlands. We requested 3751 cohort participants to consider registering for brain donation with NBB-Psy. This concerned patients with at least one of the seven following DSM-IV diagnoses: SCZ, BD, MDD, OCD, PTSD, ASD and ADHD, first-degree relatives, and healthy control subjects. Table 1 shows the number of approached participants per diagnosis group. As the OCD and PTSD cohort studies did not include any relatives, the number of relatives of OCD and PTSD patients approached is zero.

**Table 1:** The number of approached patients, relatives and controls

	SCZ	BD	MDD	OCD	PTSD	Controls
Patients	39	947	1139	493	265	
Relatives	105	262	64	0	0	
Controls						465

Participants of the ASD and ADHD cohorts were families of which the patients were minors. Within these cohorts we only approached the parents, since minors are not eligible for registration. Therefore, we cannot present registration rates of patients with ASD and ADHD.

### Procedures

With the principal investigator (PI) of each cohort study, we agreed to inform all adult cohort participants individually about NBB-Psy by means of a letter from the PI explaining the collaboration with NBB-Psy. We attached the NBB-Psy brochure, which describes why brain research of psychiatric disorders is crucial, and what to expect at the time of death. We approached as many people as possible face-to-face, immediately following their visit for the cohort study. If face-to-face contact was not possible, we approached them by post. After the first approach, we contacted participants within two weeks by telephone to offer further information unless participants objected. If the participant was interested in registering as brain donor, we sent extended information and registration forms. These forms are to be signed not only by the prospective brain donor, but also by a next-of-kin. Although Dutch law does not require permission of a next-of-kin, the NBB requires it for two reasons. First, in order to prevent someone who is (temporarily) incompetent to make the decision of becoming brain donor completely by himself. Second, co-signature ensures that people close to the prospective brain donor are informed about the registration, which increases the chance that they will contact the NBB when the brain donor dies. After three months, if the participant had not registered yet, we made a reminder phone call.

For this study, we analyzed data regarding the cohort participants who were approached between August 1, 2013 and July 1, 2015. As there was a delay of, on average, 105 days between first approach and date of actual registration, we assessed the number of registrations of these participants until October 14, 2015. All NBB-Psy procedures were approved by the medical ethics committee.

### Diagnosis

In order to analyze the relation of diagnosis with the registration rate, every cohort participant was categorized as either one of the seven NBB-Psy disorders, a relative or a control subject. In all cohort studies, the diagnoses of participants were confirmed by trained research assistants using one or multiple of the following instruments: Structured Clinical Interview for DSM-IV (SCID-I), Mini International Neuropsychiatric Interview (M.I.N.I.) plus, Comprehensive Assessment of Symptoms and History (CASH), or Composite International Diagnostic Interview (CIDI).

### Statistical analysis

Statistical analyses were carried out using SPSS 22.0. We assessed the registration percentages per group (SCZ, BD, MDD, OCD, PTSD, relatives, controls). We investigated whether age, gender, method of approach, and diagnosis affected brain-donor registration percentages. One single logistic model was used with dummy coding for the diagnostic groups relative to the controls with ‘registered as brain donor’ (yes or no) as outcome in order to compare: 1. BD versus controls, 2. MDD versus controls, 3. OCD versus controls, 4. PTSD versus controls, and 5. healthy relatives versus controls. The group SCZ was too small (*n* = 39 approached and *n* = 1 registered) to be included in the analyses. Approach method (face-to-face versus post) was a determinant and age and gender covariates. Assumptions of homogeneity of variance were checked.

We used the forward stepwise conditional method as implemented in SPSS to find the best predictive model for brain-donor registration.

## Results

### Demographic measures

We approached 3751 cohort participants. Ninety-five percent (*n* = 3567) accepted the NBB-Psy brochure (info1). Thirty-nine percent (*n* = 1446) of all the approached participants expressed a positive attitude towards brain donation and were willing to receive detailed information and registration forms (info2). Thirteen percent (*n* = 485) of the approached cohort participants registered as brain donor. During the period under study (August 2013 – October 2015), three participants withdrew their consent and five passed away and underwent autopsy. Table 2 shows the numbers for each psychiatric disorder.

**Table 2:** the approach of cohort participants, by disorder

Disorder	Cohort	Approached (n)	Average age (y)	Male	Accepted info1	Permission to phone	Info2	Registered
SCZ	GROUP	39	37	82%	n/a^b^	100%	23%	3%
BD	BiG, DIADE	947	53	46%	99%	95%	59%	25%
MDD	NESDA, NESDO, ECT	1139	54	31%	90%	84%	37%	9%
OCD	AMC regular & DBS, NOCDA	493	48	46%	95%	82%	21%	5%^c^
PTSD	BEPP/EMDR, BioMap, Booster, Paroxetine/CGT, PO	265^a^	48	52%	n/a	57%	16%	6%
Family	GROUP, BiG, NESDA	403	61	45%	100%	94%	39%	17%
Control	GROUP, BiG, NESDA, NESDO, Booster, DIADE	465	53	39%	92%	88%	32%	8%
Total		3751	53	41%	95%	86%	39%	13%

NESDA = The Netherlands Study of Depression and Anxiety. NESDO = The Netherlands study of Depression in the Elderly. ECT = Depressed patients treated with electroconvulsive therapy. BiG = Bipolar Genetics. DIADE = Diagnostic Imaging of Affective Disorders using Emotion Processing. GROUP = Genetic Risk and Outcome in Psychosis. AMC regular = Patients who had a diagnostic intake or were treated for OCD at the Academic Medical Center in Amsterdam. AMC DBS = Patients with OCD who underwent deep brain stimulation at the AMC. NOCDA = The Netherlands OCD Association Study: identifying risk factors for chronicity in the course of obsessive-compulsive disorder. Booster = The effect of oxytocin on brain activity in police officers. Paroxetine/CGT = Randomized controlled trial ‘the effectiveness of paroxetine versus trauma-focused cognitive behavioral therapy in the treatment of PTSD’. BioMap = The ‘biological markers for PTSD’ study. BEP/EMDR = ‘Effectiveness and efficiency of Eye Movement Desensitization and Reprocessing therapy (EMDR) versus Brief Eclectic Psychotherapy (BEP) in the treatment of PTSD’. PO = Police officers who have been treated for PTSD at the Academic Medical Center

^a^30% (*n* = 40) of BEPP/EMDR participants could not be reached by phone, possibly due to outdated contact information

^b^n/a = non applicable, because info1 was sent by post

^c^Those treated with Deep Brain Stimulation (*n* = 10) registered significantly more often (30%)

Two hundred and four participants were excluded from the analyses: SCZ patients were excluded as a group because the number of SCZ participants approached (*n* = 39) and registered (*n* = 1) was too low; of 4% of the participants (*n* = 141) age was unknown and 0.6% (*n* = 24) of the participants actively approached us (therefore, method of approach could not be analyzed). Exclusion of these subjects resulted in a sample of 3547 participants included in the analysis.

### Group and likelihood of registration

**Fig. 1:**
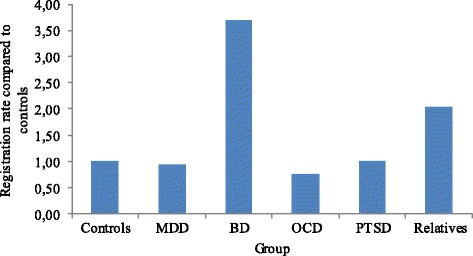
Likelihood of registration per diagnosis, with healthy control subjects as comparison

The likelihood of a patient with BD to register as a brain donor was 3.70 times higher than that of a control subject (β = 1.309, W = 42.81, *p* < 0.001). For a healthy relative the likelihood was 2.04 times higher (β = 0.710, W = 9.62, *p =* 0.002). The likelihood of brain-donor registration for MDD (β = −0.067, SE = 0.20, W = 0.11, *p* = 0.74), OCD (β = −0.284, SE = 0.27, W = 1.08, *p* = 0.30) and PTSD (β = −0.397, SE = 0.42, W = 0.92, *p* = 0.34) patients was similar to control subjects. See Fig. 1.

### Age and likelihood of registration

**Fig. 2:**
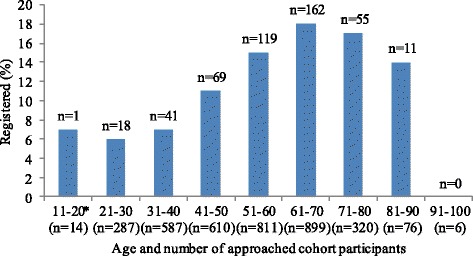
Percentage of cohort participants registered as brain donor, by age group. * Age 18-20

The likelihood of someone registering as a brain donor increased with age at least until the age of 70 (β = 0.024, W = 40.00, *p* < 0.001). Figure 2 shows the registration percentages by age group.

### Gender and likelihood of registration

There was no effect of gender on the likelihood of registering as brain donor: (β = −0.007, W = 0.004, *p* = 0.95).

### Approach method and likelihood of registration

Participants who were approached face to face registered 1.30 times more often compared to participants approached by post, which was a significant difference (β = 0.25, W = 3.90, *p* = 0.048).

### Best predictive model for brain-donor registration

The forward conditional method of logistic regression shows that the model, which best predicts the likelihood of a cohort participant registering as a brain donor includes the variables: age, method of first approach, and diagnostic group and explained around 10% of the variation (R^2^ (Nagelkerke). = 0.102).

## Discussion

In the present study, we asked psychiatric cohort participants to consider brain donation and we investigated the influence of gender, age, diagnosis, and method of approach on the willingness to sign up as brain donor. The overall registration rate was 13%, which is somewhat lower than we hypothesized (15-20%), and indeed lower than those reported for neurological cohorts (31% to 85% [6–9]). BD patients and relatives, however, did register at the anticipated rate (resp. 25% and 17%). Surprisingly, they registered more often compared to all other groups, which may be due to the fact that BD patients often articulate that their illness causes high burden, and, combined with significant genetic load of the disorder, BD patients are eager to participate in (postmortem) research. Although our sample was small, within the OCD group, patients treated with DBS registered significantly more often than other patients with OCD. This may also be due to the high burden of this DBS group, who has the most severe and therapy-resistant form of OCD. In addition, DBS patients have electrodes in their brains, which may make the link to (postmortem) brain research more logical. We expect a similar mechanism in the ECT-depression cohort, which we recently started to recruit.

In line with our hypothesis, participants, initially approached face-to-face, registered significantly more often compared to those initially approached by post. This finding also fits well with previous studies [6–8].

The likelihood of someone registering as brain donor increased with age, which is in accordance with the results of Kaye and colleagues, who found that age was positively correlated to the rate of brain-donation consent among healthy elderly participants of a longitudinal study on successful aging [5]. In absolute numbers, more females than males registered as brain donor, similar to an Australian brain donation program [10, 11]. However, we approached more women than men, so the percentage of women and men who registered was not significantly different, which is in accordance with previous studies [5, 8]. A limitation of the present study is that we were not able to perform analyses on the SCZ group due to the small sample size, nor on relatives of OCD or PTSD patients, as they were not included in the cohort studies. In addition, we could not include ASD and ADHD in our analyses because we did not have data on adult cohort participants with these diagnoses. Finally, we note that the numbers presented here concern prospective brain donors. In the period under study, three registered cohort participants withdrew their consent on second notice. The experience of the NBB is that only very few people withdraw their consent. In addition, it hardly ever occurs that the NBB is not notified in time when the donor passes away, probably as NBB asks for co-signing by a next of kin. Thus, the expectation is that the number of registered donors is almost equal to the number of autopsies that will take place in the future.

## Conclusions

In conclusion, our program resulted in a steep increase in the number of registered prospective brain donors with psychiatric diagnoses. In 2010, 149 patients with a psychiatric disorder were registered at the NBB and in October 2016, this number had risen to 1149 and equaled the number of registrations of subjects with a neurological disease.

Our results provide evidence that, when starting a prospective brain donor program, one should aim at a face-to-face approach and a focus on older people, regardless of gender. The steep increase in psychiatric brain donors will result in a huge expansion of the amount of excellently phenotyped psychiatric brain tissue available for research, which will ultimately improve our understanding of the pathophysiology of psychiatric disorders and will create the possibility to develop better treatment strategies, resulting in a higher quality of life for people with psychiatric disorders.

## Abbreviations

BEP/EMDR: ‘Effectiveness and efficiency of Eye Movement Desensitization and Reprocessing therapy (EMDR) versus Brief Eclectic Psychotherapy (BEP) in the treatment of PTSD’
Booster: The effect of oxytocin on brain activity in police officers. Paroxetine/CGT = Randomized controlled trial ‘the effectiveness of paroxetine versus trauma-focused cognitive behavioral therapy in the treatment of PTSD’
ADHD: Attention Deficit Hyperactivity Disorder
AMC DBS: Patients with OCD who underwent deep brain stimulation at the AMC
AMC regular: Patients who had a diagnostic intake or were treated for OCD at the Academic Medical Center in Amsterdam
ASD: Autism spectrum disorder
BD: Bipolar disorder
BiG: Bipolar Genetics
BioMap: The ‘biological markers for PTSD’ study
DIADE: Diagnostic Imaging of Affective Disorders using Emotion Processing
ECT: Depressed patients treated with electroconvulsive therapy
GROUP: Genetic Risk and Outcome in Psychosis
MDD: Major depressive disorder
NBB-Psy: Netherlands Brain Bank for Psychiatry
NESDA: The Netherlands Study of Depression and Anxiety
NESDO: The Netherlands study of Depression in the Elderly
NOCDA: The Netherlands OCD Association Study: identifying risk factors for chronicity in the course of obsessive-compulsive disorder
OCD: Obsessive-compulsive disorder
PO: Police officers who were treated for PTSD at the Academic Medical Center
PTSD: Post-traumatic stress disorder
SCZ: Schizophrenia
